# Diagnostic Aspects of Vitamin D: Clinical Utility of Vitamin D Metabolite Profiling

**DOI:** 10.1002/jbm4.10581

**Published:** 2021-12-03

**Authors:** Glenville Jones, Martin Kaufmann

**Affiliations:** ^1^ Department of Biomedical and Molecular Sciences Queen's University Kingston Canada

**Keywords:** 1,24,25‐(OH)_3_D_3_, 24,25‐(OH)_2_D_3_, CHRONIC KIDNEY DISEASE, HYPERCALCEMIA, LC‐MS/MS, RICKETS, VITAMIN D METABOLITE RATIO, VITAMIN D METABOLLITE PROFILING, VITAMIN D METABOLOME

## Abstract

The assay of vitamin D that began in the 1970s with the quantification of one or two metabolites, 25‐OH‐D or 1,25‐(OH)_2_D, continues to evolve with the emergence of liquid chromatography tandem mass spectrometry (LC‐MS/MS) as the technique of choice. This highly accurate, specific, and sensitive technique has been adopted by many fields of endocrinology for the measurement of multiple other components of the metabolome, and its advantage is that it not only makes it feasible to assay 25‐OH‐D or 1,25‐(OH)_2_D but also other circulating vitamin D metabolites in the vitamin D metabolome. In the process, this broadens the spectrum of vitamin D metabolites, which the clinician can use to evaluate the many complex genetic and acquired diseases of calcium and phosphate homeostasis involving vitamin D. Several examples are provided in this review that additional metabolites (eg, 24,25‐(OH)_2_D_3_, 25‐OH‐D_3_‐26,23‐lactone, and 1,24,25‐(OH)_3_D_3_) or their ratios with the main forms offer valuable additional diagnostic information. This approach illustrates that biomarkers of disease can also include metabolites devoid of biological activity. Herein, a case is presented that the decision to switch to a LC‐MS/MS technology permits the measurement of a larger number of vitamin D metabolites simultaneously and does not need to lead to a dramatic increase in cost or complexity because the technique uses a highly versatile tandem mass spectrometer with plenty of reserve analytical capacity. Physicians are encouraged to consider adding this rapidly evolving technique aimed at evaluating the wider vitamin D metabolome toward streamlining their approach to calcium‐ and phosphate‐related disease states. © 2021 The Authors. *JBMR Plus* published by Wiley Periodicals LLC on behalf of American Society for Bone and Mineral Research.

## Introduction

**Fig. 1:**
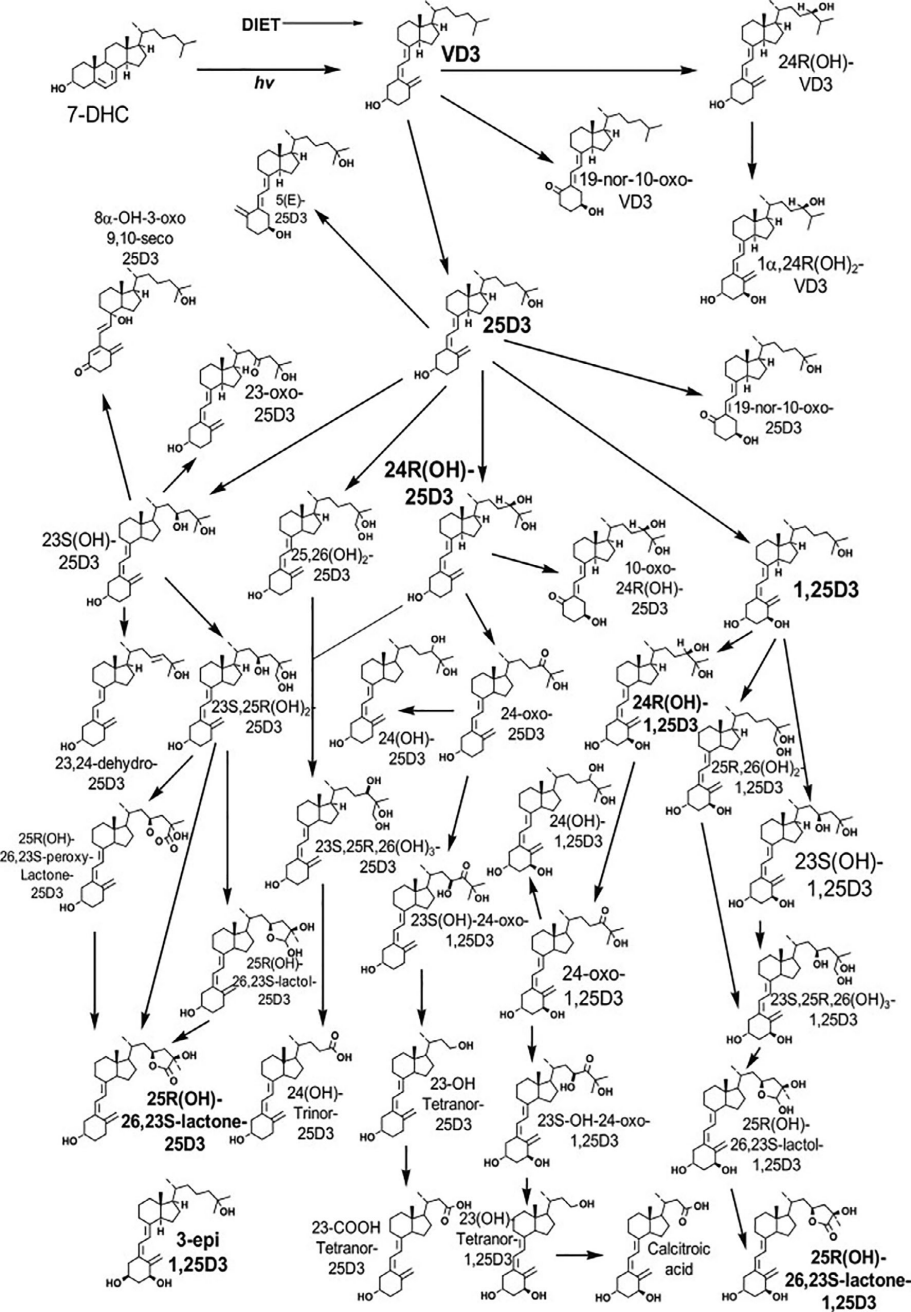
Vitamin D metabolism as depicted in 2011 by Anthony Norman (taken from Mizwicki et al.^(^ ^5^ ^)^).

The legacy of Dr Anthony Norman will always be that he initiated the highly productive and successful Vitamin D Workshops that have now continued for around five decades. However, his laboratory also made significant basic science contributions to vitamin D research by championing studies of vitamin D metabolism in the area of 24‐hydroxylation,^(^ ^1^ ^)^ intestinal calcium transport,^(^ ^2^ ^)^ vitamin D analogs,^(3^ ^)^ and rapid actions of vitamin D.^(^ ^4^ ^)^ His reviews always brought to our attention the large number of metabolites identified to date by creating complex pathway diagrams, one of which is depicted in Fig. 1.^(^ ^5^ ^)^ Over the past few decades, clinicians have largely dismissed most of these metabolites as pathway intermediates or considered them biologically inactive and therefore irrelevant to disease states and this has resulted in clinicians focusing on the pathway to the active form, 1,25‐(OH)_2_D_3_.

Because of the presence of vitamin D_2_ in pharmaceutical preparations and food supplements, the analyst must design methods to accurately measure metabolites of both vitamin D_2_ and vitamin D_3_ in serum. As a consequence, most clinical chemistry laboratories measure only a limited number of metabolites: total 25‐OH‐D (25‐OH‐D_3_ and its vitamin D_2_ equivalent, 25‐OH‐D_2_) as well as total 1,25‐(OH)_2_D (1,25‐(OH)_2_D_3_ + 1,25‐(OH)_2_D_2_). Furthermore, many clinical investigators have reduced “vitamin D assay” with measuring just total 25‐OH‐D. In July 2021, Vitamin D External Quality Assessment Scheme (DEQAS), the global vitamin D external quality assessment scheme, documented 430 clinical and research laboratories monitoring their own performance in 25‐OH‐D assays, whereas there were only 117 laboratories monitoring 1,25‐(OH)_2_D_3_ assays,^(6^ ^)^ the data suggesting that 75% of such laboratories only perform one type of vitamin D assay. Nevertheless, the current focus on measuring just one or two metabolites is understandable given that a case has been made by others that 25‐OH‐D is the best indicator of vitamin D status, whereas 1,25‐(OH)_2_D_3_ is perceived as a measure of abnormal vitamin D metabolism in certain disease states.^(^ ^7^ ^)^

**Fig. 2:**
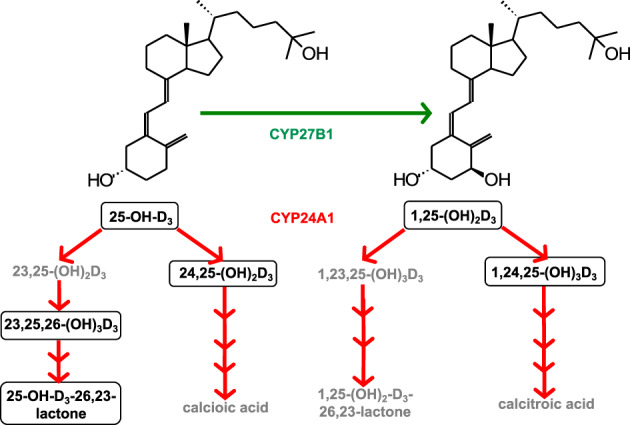
A much simplified version of vitamin D metabolism based upon those metabolites that could be used to help the physician diagnose calcium‐ and phosphate‐related diseases. Currently detectable circulating metabolites are shown in outlined boxes (modified from Kaufmann et al.^(^ ^8^ ^)^).

However, these authors believe that the emergence of liquid‐chromatography‐tandem mass spectrometry (LC‐MS/MS), as the most accurate and versatile tool in the assay of vitamin D, has created the possibility of routinely measuring many more vitamin D metabolites, including inactive ones, because they represent valuable biomarkers of dysfunctional vitamin D metabolism and therefore could be indicators of human disease. In our simplified 2021 version (Fig. 2) of Dr Norman's expansive metabolic picture, we would now include 24,25‐(OH)_2_D_3_, 25‐OH‐D_3_‐26,23‐lactone, and 1,24,25‐(OH)_3_D_3,_^(^ ^8^ ^)^ in addition to the usual 25‐OH‐D and 1,25‐(OH)_2_D as metabolites to consider in assessing clinical disease. In this review, a case will be made that most of these metabolites can be measured at the same time in multiplex LC‐MS/MS assays and that not only are the absolute levels of these compounds useful as biomarkers to the clinician but also the relative ratios of certain components can be highly indicative of disease.

## Advent of LC‐MS/MS to Revolutionize Vitamin D Assay

The assay of vitamin D has undergone a number of changes over the past five decades. On the one hand, assay of 25‐OH‐D or 1,25‐(OH)_2_D has evolved from competitive binding assays that were based upon binding to vitamin D binding globulin (VDBP) or vitamin D receptor (VDR) proteins^9^, ^10^ ^)^ to use of commercial antibody‐based kits employing proprietary antibodies that detect both D_3_ and D_2_ metabolites.^(^ ^11^, ^12^ ^)^ On the other hand, high‐pressure liquid chromatography using columns to separate D_2_ and D_3_ metabolites followed by UV_265nm_ detection^(^ ^13^ ^)^ has evolved into LC‐MS/MS employing similar chromatographic techniques but with detection based upon tandem mass spectrometry, taking advantage of the different molecular masses of vitamin D metabolites.^(^ ^14^ ^)^ Currently, there are many commercially available, antibody‐based methods for both 25‐OH‐D and 1,25‐(OH)_2_D that are quick and convenient, but based upon real‐world DEQAS user data, these methods have an average bias of about ±10% to 15%, which can exceed that limit, especially when the sample contains significant 25‐OH‐D_2._^(^ ^15^ ^)^ Indeed, there is also some question about whether these antibody‐based kits measure 25‐OH‐D_2_ with the same accuracy as 25‐OH‐D_3_ and in some cases whether they detect 25‐OH‐D_2_ at all.^(^ ^16^ ^)^ Not only are LC‐MS/MS methods more accurate for assay of 25‐OH‐D but also current LC‐MS/MS methods can separate and quantify many more metabolites using their chromatographic step and their identification detection based upon unique molecular masses. Furthermore, when measuring 25‐OH‐D, the bias of LC‐MS/MS procedures is often <5%, making their accuracy superior to the commercial antibody kits. The improved accuracy of LC‐MS/MS has resulted in one US governmental agency, Centers for Disease Control in Atlanta, to switch to using it for collection of National Health and Nutrition Examination Survey (NHANES) data.^(^ ^17^ ^)^ In addition, other US governmental agencies, including National Institute of Standards and Technology (NIST) and Office of Dietary Supplements (ODS),^(^ ^18^ ^)^ have issued a call for “standardization of 25‐OH‐D assays to superior levels of performance” and to accept only these “standardized assays” in research publications in scientific journals. External performance schemes such as DEQAS and College of American Pathologists (CAP) ensure continuing adherence to the highest attainable accuracy.

In the early 2000s, the first LC‐MS/MS assays measured one or two metabolites, such as 25‐OH‐D_3_ and 25‐OH‐D_2_, which enabled the computation of total 25‐OH‐D and comparison to antibody‐based methods, but it quickly became evident that the new and rapidly improving analytical and computational MS technology was capable of far more information with minimal additional effort. As a result, analysts began to measure further metabolites, such as 3‐epi‐25‐OH‐D_3_, especially since this specific epimer of the major circulating form was an “unwanted” contaminant of the 25‐OH‐D_3_ peak and, furthermore, LC columns were developed that allowed for it to be separated easily.^(^ ^19^ ^)^ NIST subsequently developed reference methods and distributed reference standards for 25‐OH‐D_3_, 3‐epi‐25‐OH‐D_3_ and 25‐OH‐D_2._^(^ ^14^, ^20^ ^)^ DEQAS routinely publishes quarterly results on all three of these analytes based upon target values determined currently by CDC‐Atlanta and using rigorous NIST reference methods.^(^ ^6^, ^14^ ^)^ DEQAS‐enrolled laboratories are thus able to assess the accuracy and quality of their methods in clinical and research studies. Another vitamin D metabolite rendered attainable by switching to LC‐MS/MS technology is 24,25‐(OH)_2_D_3_, which in the 1980s was measured by a somewhat laborious combination of chromatography and a competitive binding assay with VDBP.^(^ ^21^ ^)^ The LC‐MS/MS approach is much more convenient and accurate and can be made even more (10 to 100×) sensitive by derivatization of the vitamin D metabolite with a dienophile (also known as a TAD), which adds across the cis‐triene structure, making the 24,25‐(OH)_2_D_3_ and other low‐abundance metabolites detectable down into the picogram/mL range.^(^ ^22^ ^)^ Since the strategy of derivatization makes it feasible to selectively label all serum vitamin D metabolites equally efficiently at the same time, it has raised the possibility of detecting and accurately quantifying all metabolites simultaneously. Also due to the quantitative nature of the derivatization step, it is possible to predict the multiple reaction monitoring (MRM) mass transitions required to screen for a broad range of vitamin D metabolites in a somewhat untargeted manner, ushering in the concept of vitamin D metabolomics.

**Fig. 3:**
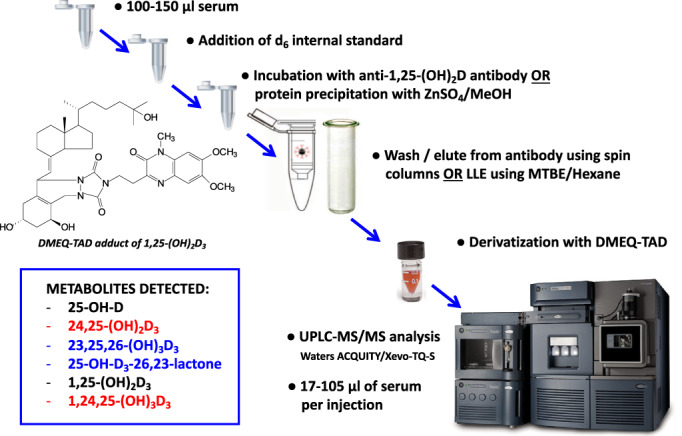
Steps in the liquid chromatography tandem mass spectrometry (LC‐MS/MS) of vitamin D metabolites when using a derivatization technique and an anti‐1,25‐(OH)_2_D_3_ antibody to detect low‐abundance forms.

In 2014, our laboratory published a LC‐MS/MS method that uses DMEQ‐TAD to derivatize the vitamin D metabolites and measures seven different vitamin D forms in a 25 to 100 μL serum sample in a single chromatographic run^(^ ^23^ ^)^ (Fig. 3). This procedure was developed to enable measurement of vitamin D metabolites in limited pediatric serum samples from idiopathic infantile hypercalcemia (IIH) patients and have even applied it to individual mice in animal studies, rather than having to pool serum from several animals.^(^ ^24^, ^25^ ^)^ Accurate assay still depends upon the availability of pure, deuterium‐labeled internal standards, many of which can be purchased commercially, as well as calibration solutions for each analyte. In some cases (eg, 25‐OH‐D_3_‐26,23‐lactone), deuterium‐labeled internal standards are not readily available and must be chemically synthesized by research‐based collaborators, but a demand for these to be made commercially usually creates a market for some company to fill.

A further development in the LC‐MS/MS of the vitamin D metabolome is the use of some modification of the technique to measure the hormone 1,25‐(OH)_2_D_3_. A decade ago, Dr Andrew Hoofnagle's group^(^ ^26^ ^)^ introduced a purification step with 1,25‐(OH)_2_D_3_ antibodies to immuno‐purify 1,25‐(OH)_2_D_3_ and 1,25‐(OH)_2_D_2_ from the contaminating lipids and other vitamin D metabolites using one of the commercially available 1,25‐(OH)_2_D_3_ antibodies. Our experience is that such antibodies also purify 1,24,25‐(OH)_3_D_3_, the presumed catabolic metabolite of 1,25‐(OH)_2_D_3_, thereby facilitating the quantification of both 1,25‐(OH)_2_D_3_ and 1,24,25‐(OH)_3_D_3_ in the picogram/mL range in 150 to 200 μL serum, in addition to the seven metabolites discussed above (Fig. 3). The resultant LC‐MS/MS technique has now been applied to and validated in both clinical and animal studies.^(^ ^27^, ^28^ ^)^

Although the sensitivity and selectivity of mass spectrometry is unparalleled, the Achilles heel of the mass spectrometry step is its inability to differentiate between isomers with the same molecular mass, unless they differ significantly in structure. A number of combinations of isomers in the vitamin D metabolome have been reported with the same molecular mass. For example, the dihydroxylated metabolites 1,25‐(OH)_2_D_3_, 23,25‐(OH)_2_D_3_, 24,25‐(OH)_2_D_3_, and 25,26‐(OH)_2_D_3_ all possess the same molecular mass and in many cases share a similar structure. If it were not for the ability to chromatographically separate these metabolites during the LC portion of LC‐MS/MS, specific measurement of these individual metabolites would not be possible by mass spectrometry alone. Specific measurement of other metabolite pairs possessing identical molecular masses and requiring baseline resolution in the chromatography step before mass spectrometry analysis include 1,24,25‐(OH)_3_D_3_ and 23,25,26‐(OH)_3_D_3_; 25‐OH‐D_3_‐26,23‐lactone and 24,25‐(OH)_2_D_2_; and most notably, 25‐OH‐D_3_ and 3epi‐25‐OH‐D_3._^(^ ^19^ ^)^ It should be noted that 1‐hydroxylated metabolites all possess a unique structural fragment from the A‐ring portion of the molecule that helps to differentiate their mass spectral properties from their non‐1‐hydroxylated counterparts and which also offers additional sensitivity when used for quantification purposes.

## Why Measure 24,25‐(OH) _2_D_3_ , 25‐OH‐D_3_ ‐26,23‐lactone, and 1,24,25‐(OH) _3_D_3_ ?

All three metabolites are products of CYP24A1, formerly known as the 25‐OH‐D_3_‐24‐hydroxylase. This enzyme was a central research focus of Dr Norman, who believed that the enzyme possessed not only a catabolic role resulting in the inactivation of vitamin D but also an anabolic function generating metabolites involved in calcification.^(^ ^1^ ^)^ Although it is fair to say that Dr Norman's theory was highly controversial in its time, more recent data from Dr Rene St‐Arnaud's laboratory working on the CYP24A1 knockout mouse has made a strong case for an anabolic role for 24‐hydroxylated metabolites in bone fracture repair.^(^ ^29^, ^30^ ^)^ Nevertheless, the prevailing dogma is that CYP24A1 plays a mainly catabolic role in the inactivation of vitamin D, producing calcitroic acid from 1,25‐(OH)_2_D_3_^(^ ^31^, ^32^, ^33^ ^)^ and calcioic acid from 25‐OH‐D_3._^(^ ^34^ ^)^

**Fig. 4:**
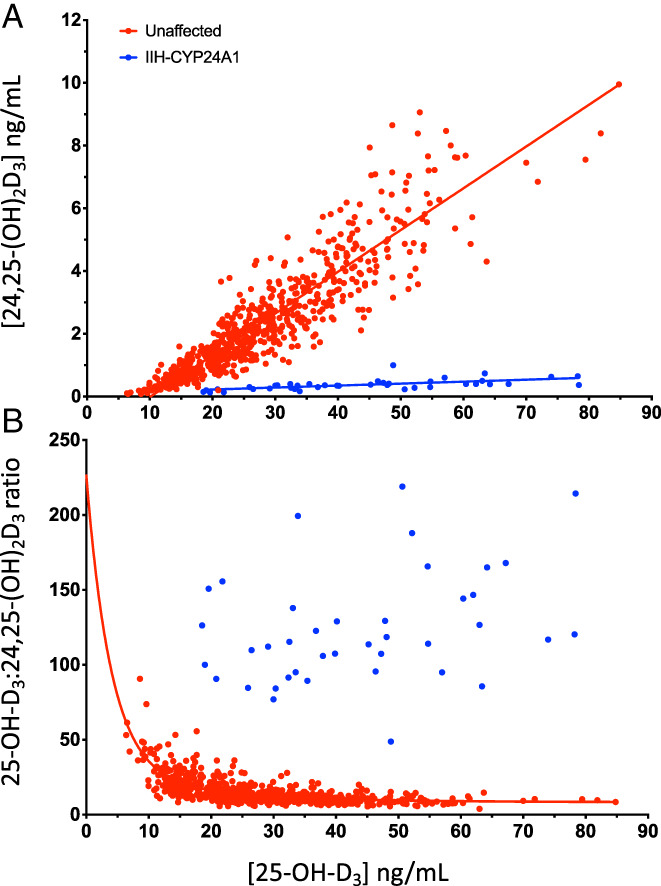
(*A*) The relationship between serum 24,25‐(OH)_2_D_3_ and serum 25‐OH‐D_3_ in normal human individuals given a range of supplements of vitamin D_3_ between 400 and 4800 IU vitamin D_3_/d for 2 years (in red; using data from Kaufmann et al.^(^ ^23^ ^)^) compared with the same relationship observed in patients with IIH due to a CYP24A1 mutation. Serum 25‐OH‐D_3_ is frequently elevated in IIH by the inability to catabolize vitamin D (in blue; using data from Molin et al.^(^ ^79^ ^)^ and Kaufmann et al.^(^ ^27^ ^)^). (*B*) The relationship between the serum 25‐OH‐D_3_/24,25‐(OH)_2_D_3_ VMR ratio and serum 25‐OH‐D_3_ in the same normal individuals receiving vitamin D_3_ (in red) and IIH patients (in blue). Note the 10‐fold increase in the ratio in IIH patients.

**Fig. 5:**
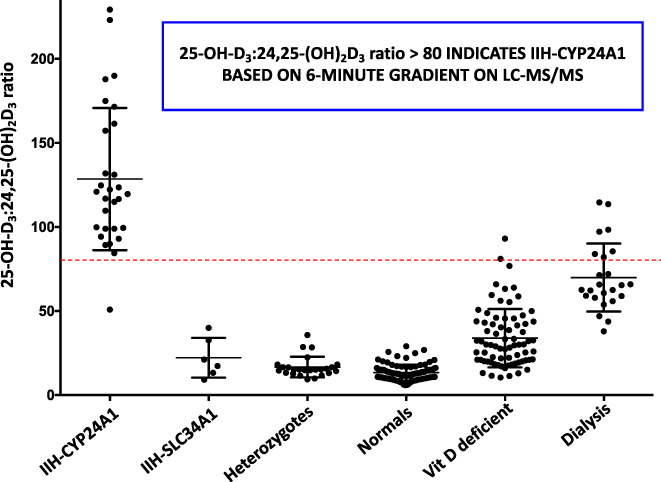
The serum 25‐OH‐D_3_/24,25‐(OH)_2_D_3_ ratio (VMR) in various patient groups (from Kaufmann et al.^(^ ^27^ ^)^). Patients with biallelic mutations of CYP24A1 show elevated VMRs above 80, while heterozygous relatives of IIH patients and normal individuals with 25‐OH‐D_3_ >20 ng/mL have VMR values in the normal range of 5 to 25 ng/mL. Individuals with 25‐OH‐D_3_ <20 ng/mL and classified as vitamin D–deficient as well as stage 5 CKD patients on dialysis show an elevated VMR above the normal range of 5 to 25 ng/mL.

Consequently, there is an overwhelming case to be made for the clinical utility of measuring 24,25‐(OH)_2_D_3_ in hypercalcemic conditions such as idiopathic infantile hypercalcemia (IIH), where there is a mutation in the *CYP24A1* gene that results in a defective CYP24A1 enzyme and little or no production of 24,25‐(OH)_2_D_3_ or other 24‐hydroxylated metabolites such as 25‐OH‐D_3_‐26,23‐lactone and 1,24,25‐(OH)_3_D_3._^(^ ^8^ ^)^ Since 24,25‐(OH)_2_D_3_ is the most abundant of these 24‐hydroxylated forms, its measurement has become the basis of the screening test for IIH in clinical practice. The serum level of 24,25‐(OH)_2_D_3_ is 10‐fold lower than control levels and justifies performing the genetic testing to confirm the IIH diagnosis^(^ ^23^, ^35^ ^)^ (Fig. 4*A*). However, one complication of this conclusion is that serum 24,25‐(OH)_2_D_3_ can also be very low in individuals with lower vitamin D nutritional status and not due to the presence of a CYP24A1 mutation. Here, a further advantage of LC‐MS/MS of the complete vitamin D metabolome is namely calculation of vitamin D metabolite ratios (VMRs), such as the ratio of 25‐OH‐D_3_/24,25‐(OH)_2_D_3_^(^ ^36^ ^)^ can be exploited (Fig. 4*B*). Because serum 24,25‐(OH)_2_D_3_ varies proportionally with 25‐OH‐D_3_, the 25‐OH‐D_3_/24,25‐(OH)_2_D_3_ ratio effectively normalizes 24,25‐(OH)_2_D_3_ against the concentration of its precursor, 25‐OH‐D_3_. Accordingly, an altered ratio indicates clinically relevant changes in 24,25‐(OH)_2_D_3_ that supersede changes in vitamin D nutrition. It should be noted here that some researchers prefer to express this VMR as the 24,25‐(OH)_2_D_3_/25‐OH‐D_3_ ratio, although the two methods have identical meaning despite the changes being in opposite directions. Our preference is to express VMRs as 25‐OH‐D_3_/24,25‐(OH)_2_D_3_ simply because values are integers and easier to comprehend than fractions. Examples of the diagnostic value of the 25‐OH‐D_3_/24,25‐(OH)_2_D_3_ ratio also occur in vitamin D deficiency and in those with chronic kidney disease (CKD) (Fig. 5), where 25‐OH‐D is also low (<12 ng/mL) and where ratios can reach levels close to those found in IIH patients.^(^ ^23^, ^35^, ^36^ ^)^ Thus, awareness of absolute 25‐OH‐D_3_ concentration is important for proper clinical evaluation of the 25‐OH‐D_3_/24,25‐(OH)_2_D_3_ ratio.

It is our opinion that the utility of serum 24,25‐(OH)_2_D_3_ in vitamin D deficiency has thus far been overlooked by the clinical fraternity. Tanaka and DeLuca^(^ ^37^ ^)^ were the first to point out the reciprocal 1α‐hydroxylase (CYP27B1) and 24‐hydroxylase (CYP24A1) activity relationship observed in mammals on a vitamin D–deficient diet as the 25‐OH‐D_3_ level falls below a “deficiency threshold,” which in humans is approximately 12 ng/mL (30 nmol/L).^(^ ^38^, ^39^ ^)^ One interpretation is that the animal in this physiological state has no purpose for synthesizing 24,25‐(OH)_2_D_3_ and needs to maximize 1,25‐(OH)_2_D_3_ production to increase intestinal calcium absorption to correct the low serum calcium level caused by vitamin D deficiency. The emergence of an accurate reference method for serum 24,25‐(OH)_2_D_3_ and reference materials has allowed testing in clinical states.^(^ ^40^ ^)^

Our work has revealed that the reciprocal 1α‐ to 24‐hydroxylase balance observed by Tanaka and DeLuca in animal studies^(^ ^37^ ^)^ also applies in vitamin D–deficient humans.^(^ ^23^ ^)^ Several studies have confirmed that a serum 24,25‐(OH)_2_D_3_ (*y* axis) versus serum 25‐OH‐D_3_ (*x* axis) plot has an *x* axis intercept showing that 24,25‐(OH)_2_D_3_ levels are virtually undetectable, ie, below the lower limit of detection (LOD) at 25‐OH‐D_3_ levels <12 ng/mL. (Fig. 4*A*).^(^ ^23^, ^41^, ^42^ ^)^ Relevant here are the values for lower limit of quantitation (LLQ) and lower limit of detection (LOD) using the DMEQ‐TAD method for 24,25‐(OH)_2_D_3_, which we reported as 0.1 to 0.2 ng/mL and 0.04 ng/mL, respectively.^(23^ ^)^ However, again the fall in the absolute level of serum 24,25‐(OH)_2_D_3_ may be less precise and thus less valuable in a clinical setting than the rise in the ratio of 25‐OH‐D_3_/24,25‐(OH)_2_D_3_ in vitamin D deficiency, which will also be discussed below (Fig. 4*B*).

The clinical case to be made for studying 25‐OH‐D_3_‐26,23‐lactone and 1,24,25‐(OH)_3_D_3_ is not as strong as that for 24,25‐(OH)_2_D_3_. 25‐OH‐D_3_‐26,23‐lactone was discovered in the late 1970s and shown by Horst^(^ ^43^ ^)^ to have a very high affinity for DBP (three‐ to fivefold higher than 25‐OH‐D_3_), making it a stable metabolite in the blood with a long half‐life. It appears to have low biological activity in intestine and bone.^(^ ^44^ ^)^ Subsequent work showed it to be formed by CYP24A1 via a complex multistep pathway involving both 23‐ and 26‐hydroxylation.^(^ ^45^ ^)^ Because its synthesis is CYP24A1‐mediated and the metabolite has a high affinity for DBP, not surprising is the fact that levels of 25‐OH‐D_3_‐26,23‐lactone rise dramatically in vitamin D–intoxicated animals^(^ ^46^, ^47^ ^)^ and humans,^21^, ^23^ ^)^ leading some to speculate that this metabolite may play a role in displacing other active forms such as 1,25‐(OH)_2_D_3_ from DBP and raising their free forms to enter the nucleus and trigger VDR‐mediated transcription during hypervitaminosis D.^(^ ^48^ ^)^ New interest in 25‐OH‐D_3_‐26,23‐lactone has been raised by the observation that high levels are also found in individuals with Williams syndrome during their hypercalcemic phase.^(^ ^8^ ^)^ In the process, the levels of 25‐OH‐D_3_‐26,23‐lactone may be a valuable biomarker in distinguishing the different causes of hypercalcemia. Thus, although 25‐OH‐D_3_‐26,23‐lactone is relatively inactive, its serum levels may tell a story that measuring only active metabolites, or one metabolite alone, does not reveal the full clinically relevant story.

Another product of CYP24A1 that can now be measured by LC‐MS/MS is 1,24,25‐(OH)_3_D_3_. Again, this metabolite was discovered decades ago by Holick and colleagues,^(^ ^49^ ^)^ who showed that not only could it be made from both 1,25‐(OH)_2_D_3_ and 24,25‐(OH)_2_D_3_, but it also retained considerable biological activity, which Dr Mark Haussler's laboratory^(^ ^50^ ^)^ estimated at 40% to 50% of that of 1,25‐(OH)_2_D_3_ based upon its interaction with the VDR. Various other researchers have demonstrated its formation and biological activity in bone cells in vitro and in vivo.^(^ ^51^, ^52^, ^53^ ^)^ The molecular similarity between 1,24,25‐(OH)_3_D_3_ and 1,25‐(OH)_2_D_3_ is reinforced when one considers the fact that commercially available 1,25‐(OH)_2_D_3_ antibodies used in LC‐MS/MS immunoaffinity steps co‐purify 1,24,25‐(OH)_3_D_3_. In the 1980s, O′Riordan's laboratory used a radioimmunoassay to detect 1,24,25‐(OH)_3_D_3_ in human serum in the 10 to 20 pg/mL range.^(^ ^54^ ^)^ Recent collaborative studies by our laboratory in conjunction with a Calgary team headed by Dr David Hanley^(^ ^55^ ^)^ suggest that in the normal vitamin D replete state, serum 1,24,25‐(OH)_3_D_3_ levels are similar to those of serum 1,25‐(OH)_2_D_3_ but rise above those of 1,25‐(OH)_2_D_3_ during vitamin D supplementation. This poses the question as to whether 1,24,25‐(OH)_3_D_3_ plays any role in the biological actions of vitamin D, and its assay might be useful in certain clinical situations or maybe is just a degradation product of excess 1,25‐(OH)_2_D_3_.

In assessing whether these CYP24A1‐derived 24‐hydroxylated vitamin D metabolites are worth measuring clinically, one must also consider that multiple gene expression studies in animal and human cells have shown that the *CYP24A1* gene, as part of the auto‐regulatory response, is the gene most dramatically induced in the whole genome by 1,25‐(OH)_2_D_3._^(^ ^56^, ^57^ ^)^ Consequently, it should come as no real surprise that the in vivo production of 24‐hydroxylated vitamin D metabolites is increased and blood levels rise when vitamin D or 25‐OH‐D is administered, especially since these 24‐hydroxylated forms have a strong affinity for DBP. A case could be made therefore that serum levels of 24,25‐(OH)_2_D_3_, 25‐OH‐D_3_‐26,23‐lactone, and 1,24,25‐(OH)_3_D_3_ are all surrogates or biomarkers of the biological action of 1,25‐(OH)_2_D_3_ on cellular gene expression.^(^ ^28^ ^)^ Because they all have an affinity for VDBP and are more stable in the blood than the hormone, they may remain elevated when the levels of 1,25‐(OH)_2_D_3_, with its short half‐life measured in hours, are declining. Moreover, this may suggest that these three 24‐hydroxylated forms could be better biomarkers than the blood levels of the hormone itself, since 1,25‐(OH)_2_D_3_ assay is notoriously labile with a half‐life in hours^(^ ^58^ ^)^ and clinically unhelpful in some situations.

## Additional Vitamin D Metabolites That Could Be Measured

Students of vitamin D metabolism and those who view Dr Norman's depiction of the complex metabolic picture in Fig. 1 will no doubt wonder about a few other vitamin D molecules that are feasible to measure along with those discussed: the parent vitamin D itself (either cholecalciferol or ergocalciferol),^(59^ ^)^ free 25‐OH‐D_3_,^(60^ ^)^ 3epi‐25‐OH‐D_3_,^14^, ^20^, ^61^ ^)^ 24,25‐(OH)_2_D_2_,^(62^ ^)^ 1,24,25‐(OH)_3_D_2_,^(63^ ^)^ and 25,26‐(OH)_2_D_3_.^(^ ^27^, ^64^ ^)^

Vitamin D_2_ and D_3_ would seem to be obvious targets and are at levels in the blood that make it easy to detect them. There are even deuterium or ^13^C‐labeled internal standards available from commercial suppliers. However, it was shown many decades ago by the laboratory of Dr Hector DeLuca that [^3^H]vitamin D made in the skin or from the diet is rapidly cleared from the bloodstream by the liver,^(65^ ^)^ making the clinical value of this volatile parameter debatable. It is conceivable that studies of the half‐life of skin synthesized vitamin D_3_ or the clearance of dietary supplements of vitamins D_2_ or D_3_ could have value, especially where liver dysfunction is documented. One such example is vitamin D–dependent rickets type 1B, where mutations of CYP2R1 result in the lack of 25‐hydroxylation and LC‐MS/MS would show a metabolic block in the conversion of vitamin D to 25‐OH‐D.^(^ ^66^ ^)^ However, these patients are extremely rare, and currently, we have limited experience with them to justify routine assay of vitamin D. However, there are other more common liver diseases^(^ ^67^ ^)^ where assessment of the vitamin D/25‐OH‐D ratio might be feasible using LC‐MS/MS.

The accurate measurement of free 25‐OH‐D_3_ has been a goal of analysts/endocrinologists for decades since the free hormone hypothesis became popular.^(^ ^67^ ^)^ But assay of a free serum molecule 25‐OH‐D (or 1,25‐(OH)_2_D) at extremely low concentrations even for LC‐MS/MS has not been achieved by this technique. Free 25‐OH‐D is still best measured by antibody‐based methods^(^ ^68^ ^)^ or by calculation using accurate measurement of total 25‐OH‐D and vitamin D–binding globulin (VDBP or DBP).^(^ ^69^ ^)^

3‐epi‐25‐OH‐D_3_ is a recently discovered metabolite^(^ ^61^ ^)^ that was first detected in clinical serum samples by LC‐MS/MS and is not detected by antibody‐based methods. It can be easily resolved from 25‐OH‐D_3_ by specific LC columns^(^ ^19^ ^)^ and in adults its level averages around 5% of the total 25‐OH‐D_3._^(^ ^23^ ^)^ Interestingly, the concentration of 3‐epi‐25‐OH‐D_3_ is much higher in newborns, reaching as high as 50% of total 25‐OH‐D_3_,^61^, ^70^ ^)^ but this slowly declines to adult levels by about 2 years of age. However, its biological function and source remain unknown and thus we do not recommend its routine measurement in clinical studies.

24,25‐(OH)_2_D_2_^(^ ^62^ ^)^ and 1,24,25‐(OH)_3_D_2_^(^ ^63^ ^)^ can be detected in serum, but they are minor metabolites of vitamin D_2_, made even more insignificant by the low use of vitamin D_2_ in clinical studies around the world. This is the main reason most analysts focus on ratios of the vitamin D_3_ metabolites such as the ratio of 25‐OH‐D_3_/24,25‐(OH)_2_D_3_ to be discussed below. Another serum metabolite that can be detected is 25,26‐(OH)_2_D_3_, but again as with 3‐epi‐25‐OH‐D_3_, its biological function is unknown and again our main purpose in LC‐MS/MS assays is to eliminate it from interfering in the accurate measurement of 24,25‐(OH)_2_D_3_ and 1,25‐(OH)_2_D_3_^(^ ^27^ ^)^

## The Added Value of Multiplexed Assays—The Complete Vitamin D Metabolome

When clinical assays for vitamin D started in the early 1970s, the clinician was content to get a single metabolite measure, namely 25‐OH‐D, to assess the vitamin status of the patient. The later addition of serum 1,25‐(OH)_2_D as a parameter to assess the hormonal form was useful but involved a separate laboratory analysis. Clinicians have been content with these two parameters for decades but now have the option of assaying a greater number of clinically relevant vitamin D metabolites by utilizing multiplexed LC‐MS/MS. What is more is that this can be achieved with just a small increase in laboratory complexity and cost^(^ ^71^ ^)^ because of the use of a single extraction and LC step, as well as multiple reaction monitoring using different metabolite fragments to detect all metabolites present in the serum. Consequently, the type of LC‐MS/MS assay depicted in Fig. 3 has the capability of measuring approximately 10 metabolites, including the important biologically active metabolites of both vitamin D_2_ and D_3_ simultaneously, provided that appropriate deuterated internal standards are employed.

The simultaneous assay of the “complete” clinically relevant vitamin D metabolome also allows for the calculation of vitamin D metabolite ratios, the most studied ratio being that of 25‐OH‐D_3_/24,25‐(OH)_2_D_3_. This was first proposed by Dr Reinhold Vieth,^(36^ ^)^ who, using a limited number of samples, pointed out that the two metabolites showed a virtually linear correlation as 25‐OH‐D_3_ increased. In 2014, our laboratory applied a multiplexed LC‐MS/MS approach to study serum samples from pre‐ and postmenopausal women with osteopenia given vitamin D_3_ supplements (400 IU to 4800 IU/d) for 2 years.^(^ ^23^ ^)^ We showed that serum 25‐OH‐D_3_ ranged from 20 to 80 ng/mL depending on the dose of vitamin D_3_ and that serum 24,25‐(OH)_2_D_3_ increased linearly with the increase in 25‐OH‐D_3_, as Vieth and colleagues^(^ ^36^ ^)^ had found. Our principal objective was to study the normal range of serum 24,25‐(OH)_2_D_3_ values over a wide range of 25‐OH‐D_3_ but also to observe the ratio of 25‐OH‐D_3_/24,25‐(OH)_2_D_3_ and how that ratio changed over time as the study included multiple samples from the same individuals. Not only is the 25‐OH‐D_3_/24,25‐(OH)_2_D_3_ ratio relatively stable in individuals over time (3 years in ref),^(^ ^55^ ^)^ but also the range of values in the whole population remains relatively narrow between a ratio value of 5 to 25 (Table 1). Values of this 25‐OH‐D_3_/24,25‐(OH)_2_D_3_ ratio >25 suggest CYP24A1 dysregulation.

**Table 1:** Means and Normal Ranges of Vitamin D Metabolites

	No.	25‐OH‐D_3_	24,25‐(OH)_2_D_3_	Ratio	25‐OH‐D_3_‐26,23‐lactone	1α,25‐(OH)_2_D_3_	1α,24,25‐(OH)3D3
		(ng/mL)	(ng/mL)	25‐OH‐D_3_/24,25‐(OH)_2_D_3_	(ng/mL)	(pg/mL)	(pg/mL)
25‐OH‐D >20	84	35.4 ± 19.4	2.88 ± 1.90	12.8 ± 4.5	0.089 ± 0.069	32.0 ± 10.8	9.1 ± 3.8
25‐OH‐D <20	79	11.7 ± 5.2	0.47 ± 0.30	31.9 ± 15.9	0.038 ± 0.021	36.5 ± 12.6	5.7 ± 3.4
25‐OH‐D‐all	163	24.7 ± 18.9	1.79 ± 1.86	21.4 ± 14.7	0.070 ± 0.06	33.8 ± 11.8	7.6 ± 3.9
95% interval	163	5.6–70.7	0.15–5.60	7.7–55.1	0.015–0.195	16.2–53.9	2.1–15.5

Values were taken from normal individuals with 25‐OH‐D >20 or <20 ng/mL from studies in Nebraska, USA; France; and Germany, published by Kaufmann et al.^(^ ^8^ ^)^

There are at least three reasons for a high 25‐OH‐D_3_/24,25‐(OH)_2_D_3_ VMR value:

1. IIH, where the *CYP24A1* gene is mutated and defective;
2. vitamin D deficiency, where parathyroid hormone (PTH) secretion turns off the *CYP24A1* gene; and
3. CKD, where there is progressive loss of renal function and/or hormonal dysregulation.

With reason 1, the 10‐fold rise in the 25‐OH‐D_3_/24,25‐(OH)_2_D_3_ VMR in IIH is dramatic and a value of >80 (Figs. 4 *B* and 5) serves to immediately provide an indicator that the patient has a genetic CYP24A1 defect, since it simultaneously takes into consideration the level of substrate 25‐OH‐D. In our hands, this screening tool is 100% accurate at predicting those who have biallelic mutations of the *CYP24A1* gene.^(^ ^23^, ^35^ ^)^

Reasons 2 and 3 involve acquired diseases and the 25‐OH‐D_3_/24,25‐(OH)_2_D_3_ VMR rises gradually to values >25 and often doubles to a ratio of around 50 or higher but rarely reaches the value >80 attained in IIH. Thus, a VMR over the threshold >25 (Figs. 4 *B* and 5) is a sensitive indicator of vitamin D deficiency and could be a very useful additional parameter for the pediatrician/clinician using serum 25‐OH‐D <12 ng/mL and a PTH assay to assess vitamin D deficiency.^(^ ^41^, ^42^ ^)^ In CKD, the pathogenesis involved is different, suggesting a gradual loss or downregulation of 24‐hydroxylation of 25‐OH‐D_3_,^(72^ ^)^ but the consequence is the same and the 25‐OH‐D_3_/24,25‐(OH)_2_D_3_ VMR often rises to an even greater degree than in simple vitamin D deficiency. A group of nephrologists who reviewed the case for this 25‐OH‐D_3_/24,25‐(OH)_2_D_3_ VMR concluded that this is a useful tool for assessing progression of the loss of 24‐hydroxylation observed in stages 3 and 4 of CKD.^(^ ^73^ ^)^ One important point to make relative to renal disease is that the 25‐OH‐D_3_/24,25‐(OH)_2_D_3_ VMR is independent of the concentration of vitamin D binding globulin^(^ ^74^ ^)^ presumably because both metabolites have equal affinity for the plasma transporter.^(^ ^75^ ^)^

Other researchers have promoted the value of the 25‐OH‐D_3_/24,25‐(OH)_2_D_3_ VMR with changes in bone density and fracture risk. A recent study by Hoofnagle's group^(^ ^76^ ^)^ showed that a 50% lower 24,25‐(OH)_2_D_3_/25‐OH‐D_3_ VMR was associated with a 0.3% more rapid decline in total hip bone mineral density (BMD) in a group of 70‐ to 79‐year‐old community‐dwelling adults. It is important to note that Hoofnagle and colleagues^(^ ^76^ ^)^ use an inverted ratio compared with that presented here, ie, 25‐OH‐D_3_/24,25‐(OH)_2_D_3_ VMR, resulting in a decline rather than a rise in their ratio and completely consistent with the changes namely a decline in 24‐hydroxylation that is observed in CKD patients.^(^ ^72^, ^73^ ^)^ In contrast, lower (absolute) 25‐OH‐D_3_ concentrations were not associated with a longitudinal change in BMD. A similar relationship between 24,25‐(OH)_2_D_3_/25‐OH‐D_3_ VMR and fracture risk was also observed in this study. It is worth noting that 23% of the studied group had an estimated glomerular filtration rate (eGFR) <60 mL/mL/1.73m^2^,^(76^ ^)^ suggesting that the underlying causes were the same as those observed in the CKD patients.^(^ ^72^, ^73^ ^)^

While other VMR ratios such as 25‐OH‐D_3_/1,25‐(OH)_2_D_3_ or 1,25‐(OH)_2_D_3_/24,25‐OH‐D_3_ might offer clinical potential, we are yet to observe their clear value in any of the studies we have been involved in. Hoofnagle presents data that argue for a possible valuable role for the 25‐OH‐D_3_/1,25‐(OH)_2_D_3_ VMR in clinical studies.^(^ ^77^ ^)^ A case can also be presented for the 1,25‐(OH)_2_D_3_/1,24,25‐(OH)_3_D_3_ VMR, which offers a similar anabolic/catabolic relationship to that found for the 25‐OH‐D_3_/24,25‐(OH)_2_D_3_ VMR in bone health.^(^ ^76^ ^)^

A further additional advantage of the comparison of vitamin D metabolites in the form of vitamin D metabolite ratios stemming from the complete LC‐MS/MS metabolome is the measurement of the vitamin D_3_ metabolite/vitamin D_2_ metabolite ratio. The most readily available ratio to the clinician from the LC‐MS/MS analysis is the 25‐OH‐D_3_/25‐OH‐D_2_ ratio, which allows the physician to assess the contribution of a patient's vitamin D_2_ dietary intake. This can come from vitamin D_2_ in plant‐derived foods (eg, irradiated yeast, mushrooms) or vitamin D_2_‐containing supplements or pharmaceuticals (particularly in the US, where these therapeutic agents are sometimes used in place of vitamin D_3_).^(^ ^78^ ^)^ Currently, analytical data suggest that serum 25‐OH‐D_2_ levels are very low except when the patient derives vitamin D from unfortified food sources alone and is not treated with vitamin D_2_. Consequently, the serum 25‐OH‐D_2_ serves as a useful biomarker that indicates whether the patient is responsive to oral vitamin D treatment.

In summary, the elevated 25‐OH‐D_3_/24,25‐(OH)_2_D_3_ ratio unequivocally identifies patients with CYP24A1 mutations. While other patients with hypercalcemia related to vitamin D possess normal ratios, such as patients with SLC34A1 mutation, hypervitaminosis D patients, and certain Williams syndrome patients, it should not be overlooked that measurement of other vitamin D metabolites and attention to absolute concentrations point to distinct metabolite profiles in each of these cases, suggesting that measurement of the complete vitamin D metabolome can aid in the differential diagnosis of vitamin D–related hypercalcemia. In a recent study,^(^ ^8^ ^)^ hypercalcemic WBS patients and other idiopathic hypercalcemia cases exhibited increased 25‐OH‐D_3_‐lactone, 23,25,26‐(OH)_3_D_3_, and 1,24,25‐(OH)_3_D_3_, while possessing very low 1,25‐(OH)_2_D_3_. One interpretation of this profile is that it indicates hypersensitive transactivation of vitamin D–dependent genes as an underlying cause of the hypercalcemia, since CYP24A1 is the most upregulated gene in response to 1,25‐(OH)_2_D_3_ hormonal action, which is consistent with increased concentration of most metabolites formed by CYP24A1. Not only does measurement of the vitamin D metabolome facilitate rapid identification of the underlying cause of hypercalcemia among known causes, but it also enables the proposal of novel mechanisms of pathogenesis that can be used to select new candidate genes.

## Disclosures

The authors state that they have no conflicts of interest.

### Peer review

The peer review history for this article is available at https://publons.com/publon/10.1002/jbm4.10581.

## References

[1] Henry HL , Norman AW . Vitamin D: metabolism and biological actions. Annu Rev Nutr. 1984;4:493‐520. 6087861 10.1146/annurev.nu.04.070184.002425

[2] Haussler MR , Myrtle JF , Norman AW . The association of a metabolite of vitamin D3 with intestinal mucosa chromatin in vivo. J Biol Chem. 1968;243:4055‐4064. 5666948

[3] Bouillon R , Okamura WH , Norman AW . Structure‐function relationships in the vitamin D endocrine system. Endocr Rev. 1995;16:200‐257. 7781594 10.1210/edrv-16-2-200

[4] Norman AW , Okamura WH , Bishop JE , Henry HL . Update on biological actions of 1alpha,25(OH)2‐vitamin D3 (rapid effects) and 24R,25(OH)2‐vitamin D3. Mol Cell Endocrinol. 2002;197:1‐13. 10.1016/s0303-7207(02)00273-3 12431790

[5] Mizwicki MT , Norman AW . Chapter 15: vitamin D sterol/VDR conformational dynamics and nongenomic actions. In Feldman D , Pike JW , Adams JD , eds. Vitamin D. 3rd ed. Waltham, MA: Academic Press; 2011 pp 271‐297.

[6] Vitamin D External Quality Assessment Scheme (DEQAS) [Internet]. Charing Cross Hospital, London. Available at: http://www.deqas.org.

[7] Holick MF . Vitamin D deficiency. N Engl J Med. 2007;357:266‐281. 17634462 10.1056/NEJMra070553

[8] Kaufmann M , Schlingmann K‐P , Berezin L , et al. Differential diagnosis of vitamin D‐related hypercalcemia using serum vitamin D metabolite profiling. J Bone Miner Res. 2021;36:1340‐1350. 33856702 10.1002/jbmr.4306

[9] Belsey R , DeLuca HF , Potts JT Jr . Competitive binding assay for vitamin D and 25‐OH vitamin D. J Clin Endocrinol Metab. 1971;33:554‐557. 4328344 10.1210/jcem-33-3-554

[10] Eisman JA , Hamstra AJ , Kream BE , DeLuca HF . 1,25‐Dihydroxyvitamin D in biological fluids: a simplified and sensitive assay. Science. 1976;193(4257):1021‐1023. 1085035 10.1126/science.1085035

[11] Hollis BW . Comparison of commercially available ^125^I‐based RIA methods for the determination of circulating 25‐hydroxyvitamin D. Clin Chem. 2000;46:1657‐1661. 11017946

[12] Hollis BW . Assessment of circulating 25(OH)D and 1,25(OH)2D: emergence as clinically important diagnostic tools. Nutr Rev. 2007;65:S87‐S90. 17867378 10.1111/j.1753-4887.2007.tb00348.x

[13] Jones G . Assay of vitamin D_2_ and D_3_, 25‐hydroxyvitamins D_2_ and D_3_ in human plasma by high pressure liquid chromatography. Clin Chem. 1978;24:287‐298. 203413

[14] Tai SS , Bedner M , Phinney KW . Development of a candidate reference measurement procedure for the determination of 25‐hydroxyvitamin D3 and 25‐hydroxyvitamin D2 in human serum using isotope‐dilution liquid chromatography‐tandem mass spectrometry. Anal Chem. 2010;82:1942‐1948. 20136128 10.1021/ac9026862 PMC2838390

[15] Carter G , Jones, J , Walker, E et al. DEQAS annual reports, 2017 [Internet]. Charing Cross Hospital, London. Available at: http://www.deqas.org.

[16] Horst RL . Exogenous versus endogenous recovery of 25‐hydroxyvitamins D2 and D3 in human samples using high‐performance liquid chromatography and the DiaSorin LIAISON Total‐D Assay. J Steroid Biochem Mol Biol. 2010;121(1–2):180‐182. 20214981 10.1016/j.jsbmb.2010.03.010

[17] Yetley EA , Pfeiffer CM , Schleicher RL , et al. NHANES monitoring of serum 25‐hydroxyvitamin D: a roundtable summary. J Nutr. 2010;140(11):2030S‐2045S. 20881084 10.3945/jn.110.121483 PMC2955879

[18] Sempos CT , Vesper HW , Phinney KW , Thienpont LM , Coates PM . Vitamin D standardization program (VDSP). Vitamin D status as an international issue: national surveys and the problem of standardization. Scand J Clin Lab Invest Suppl. 2012;243:32‐40. 22536760 10.3109/00365513.2012.681935

[19] van den Ouweland JM , Beijers AM , van Daal H . Overestimation of 25‐hydroxyvitamin D3 by increased ionisation efficiency of 3‐epi‐25‐hydroxyvitamin D3 in LC‐MS/MS methods not separating both metabolites as determined by an LC‐MS/MS method for separate quantification of 25‐hydroxyvitamin D3, 3‐epi‐25‐hydroxyvitamin D3 and 25‐hydroxyvitamin D2 in human serum. J Chromatogr B Analyt Technol Biomed Life Sci. 2014;967:195‐202. 10.1016/j.jchromb.2014.07.021 25125396

[20] Tai SS , Nelson MA , Bedner M , et al. Development of standard reference material (SRM) 2973 vitamin D metabolites in frozen human serum (high level). J AOAC Int. 2017;100:1294‐1303. 28917261 10.5740/jaoacint.17-0182

[21] Shepard RM , Horst RL , Hamstra AJ , DeLuca HF . Determination of vitamin D and its metabolites in plasma from normal and anephric man. Biochem J. 1979;182:55‐69. 227368 10.1042/bj1820055 PMC1161234

[22] Shimizu M , Kamachi S , Nishii Y , Yamada S . Synthesis of a reagent (DMEQ‐TAD) for fluorescence‐labeling of vitamin D and its use in assaying vitamin D metabolites. Anal Biochem. 1991;194:77‐81. 1867383 10.1016/0003-2697(91)90153-k

[23] Kaufmann M , Gallagher C , Peacock M , et al. Clinical utility of simultaneous quantitation of 25‐hydroxyvitamin D & 24,25‐dihydroxyvitamin D by LC‐MS/MS involving derivatization with DMEQ‐TAD. J Clin Endocrinol Metab. 2014;99:2567‐2574. 24670084 10.1210/jc.2013-4388 PMC4079315

[24] Meyer MB , Benkusky NA , Kaufmann M , et al. A kidney‐specific genetic control module in mice governs endocrine regulation of the cytochrome P450 gene Cyp27b1 essential for vitamin D_3_ activation. J Biol Chem. 2017;292:17541‐17558. 28808057 10.1074/jbc.M117.806901 PMC5655528

[25] Ryan BA , Alhani K , Sellars KB , et al. Mineral homeostasis in murine fetuses is sensitive to maternal calcitriol, but not to absence of fetal calcitriol. J Bone Miner Res. 2019;34:669‐680. 30508318 10.1002/jbmr.3642

[26] Laha TJ , Strathmann FG , Wang Z , de Boer IH , Thummel KE , Hoofnagle AN . Characterizing antibody cross‐reactivity for immunoaffinity purification of analytes prior to multiplexed liquid chromatography‐tandem mass spectrometry. Clin Chem. 2012;58:1711‐1716. 22968104 10.1373/clinchem.2012.185827 PMC3731945

[27] Kaufmann M , Morse N , Molloy BJ , et al. Improved screening test for idiopathic infantile hypercalcemia confirms residual levels of serum 24,25‐(OH)_2_D_3_ in affected patients. J Bone Mineral Res. 2017;32:1589‐1596. 10.1002/jbmr.3135 28304097

[28] Meyer MB , Benkusky NA , Kaufmann M , et al. Targeted genomic deletions identify diverse enhancer functions and generate a kidney‐specific, endocrine‐deficient *Cyp27b1* pseudo‐null mouse. J Biol Chem. 2019;294:9518‐9535. 31053643 10.1074/jbc.RA119.008760 PMC6579472

[29] St‐Arnaud R , Naja RP . Vitamin D metabolism, cartilage and bone fracture repair. Mol Cell Endocrinol. 2011;347:48‐54. 21664253 10.1016/j.mce.2011.05.018

[30] Martineau C , Naja RP , Husseini A , et al. Optimal bone fracture repair requires 24R,25‐dihydroxyvitamin D3 and its effector molecule FAM57B2. J Clin Invest. 2018;128:3546‐3557. 30010626 10.1172/JCI98093 PMC6063485

[31] Jones G , Kaufmann M , Prosser D . 25‐hydroxyvitamin D_3_‐24‐hydroxylase (CYP24A1): its important role in the degradation of vitamin D. Arch Biochem Biophys. 2012;523:9‐18. 22100522 10.1016/j.abb.2011.11.003

[32] Makin G , Lohnes D , Byford V , Ray R , Jones G . Target cell metabolism of 1,25‐(OH)_2_D_3_ to calcitroic acid: evidence for a pathway in kidney and bone involving 24‐oxidation. Biochem J. 1987;262:173‐180. 10.1042/bj2620173 PMC1133244 2818561

[33] Reddy GS , Tserng KY . Calcitroic acid, end product of renal metabolism of 1,25‐dihydroxyvitamin D3 through C‐24 oxidation pathway. Biochemistry. 1989;28:1763‐1769. 2719932 10.1021/bi00430a051

[34] Kaufmann M , Martineau C , Arabian A , Traynor M , St‐Arnaud R , Jones G . Calcioic acid: *In vivo* detection and quantification of the terminal C24‐oxidation product of 25‐hydroxyvitamin D_3_ and related intermediates in serum of mice treated with 24,25‐dihydroxyvitamin D_3_ . J Steroid Biochem Mol Biol. 2019;188:23‐28. 30553931 10.1016/j.jsbmb.2018.12.001 PMC9703456

[35] Schlingmann KP , Kaufmann M , Weber S , et al. Mutations of CYP24A1 and idiopathic infantile hypercalcemia. N Engl J Med. 2011;365:410‐421. 21675912 10.1056/NEJMoa1103864

[36] Wagner D , Hanwell HE , Schnabl K , et al. The ratio of serum 24,25‐dihydroxyvitamin D(3) to 25‐hydroxyvitamin D(3) is predictive of 25‐hydroxyvitamin D(3) response to vitamin D(3) supplementation. J Steroid Biochem Mol Biol. 2011;126:72‐77. 21605672 10.1016/j.jsbmb.2011.05.003

[37] Tanaka Y , DeLuca HF . Measurement of mammalian 25‐hydroxyvitamin D3 24R‐and 1 alpha‐hydroxylase. Proc Natl Acad Sci U S A. 1981;78:196‐199. 6972531 10.1073/pnas.78.1.196 PMC319018

[38] Ross AC , Manson JE , Abrams SA , et al. The 2011 report on dietary reference intakes for calcium and vitamin D from the Institute of Medicine: what clinicians need to know. J Clin Endocrinol Metab. 2011;96:53‐58. 21118827 10.1210/jc.2010-2704 PMC3046611

[39] Munns CF , Shaw N , Kiely M , et al. Global consensus recommendations on prevention and management of nutritional rickets. J Clin Endocrinol Metab. 2016;101:394‐415. 26745253 10.1210/jc.2015-2175 PMC4880117

[40] Tai SS , Nelson M . Candidate reference measurement procedure for the determination of (24R),25‐Dihydroxyvitamin D3 in human serum using isotope‐dilution liquid chromatography‐tandem mass spectrometry. Anal Chem. 2015;87:7964‐7970. 26171884 10.1021/acs.analchem.5b01861

[41] Wise SA , Tai SS , Nelson MA , et al. Interlaboratory comparison for the determination of 24,25‐Dihydroxyvitamin D3 in human serum using liquid chromatography with tandem mass spectrometry. J AOAC Int. 2017;100:1308‐1317. 28741469 10.5740/jaoacint.17-0183

[42] Cashman KD , Hayes A , Galvin K , et al. Significance of serum 24,25‐dihydroxyvitamin D in the assessment of vitamin D status: a double‐edged sword? Clin Chem. 2015;61:636‐645. 25710460 10.1373/clinchem.2014.234955

[43] Horst RL . 25‐OHD3‐26,23‐lactone: a metabolite of vitamin D3 that is 5 times more potent than 25‐OHD3 in the rat plasma competitive protein binding radioassay. Biochem Biophys Res Commun. 1979;89:286‐293. 475814 10.1016/0006-291x(79)90976-8

[44] Tanaka Y , Wichmann JK , Paaren HE , Schnoes HK , DeLuca HF . Role of kidney tissue in the production of 25‐hydroxyvitamin D3‐26,23‐lactone and 1 alpha, 25‐dihydroxyvitamin D3‐26,23‐lactone. Proc Natl Acad Sci U S A. 1980;77:6411‐6414. 6935655 10.1073/pnas.77.11.6411 PMC350294

[45] Sakaki T , Sawada N , Komai K , et al. Dual metabolic pathway of 25‐hydroxyvitamin D3 catalyzed by human CYP24. Eur J Biochem. 2000;267:6158‐6165. 11012668 10.1046/j.1432-1327.2000.01680.x

[46] Shephard RM , Deluca HF . Plasma concentrations of vitamin D3 and its metabolites in the rat as influenced by vitamin D3 or 25‐hydroxyvitamin D3 intakes. Arch Biochem Biophys. 1980;202:43‐53. 6249223 10.1016/0003-9861(80)90404-x

[47] Jones G , Vriezen D , Lohnes D , Palda V , Edwards NS . Side‐chain hydroxylation of vitamin D3 and its physiological implications. Steroids. 1987;49:29‐53. 2842896 10.1016/0039-128x(87)90078-x

[48] Jones G . Pharmacokinetics of vitamin D toxicity. Am J Clin Nutr. 2008;88:582S‐586S. 18689406 10.1093/ajcn/88.2.582S

[49] Holick MF , Kleiner‐Bossaller A , Schnoes HK , Kasten PM , Boyle IT , DeLuca HF . 1,24,25‐Trihydroxyvitamin D3. A metabolite of vitamin D3 effective on intestine. J Biol Chem. 1973;248:6691‐6696. 4355503

[50] Chandler JS , Pike JW , Haussler MR . Biosynthesis, purification and receptor binding properties of high specific radioactivity 1 alpha, 24(R),25‐trihydroxy‐[26,27‐methyl‐3H]‐vitamin D3. J Steroid Biochem. 1982;16:303‐310. 6281579 10.1016/0022-4731(82)90181-9

[51] Miller BE , Chin DP , Jones G . 1,25‐Dihydroxyvitamin D3 metabolism in a human osteosarcoma cell line and human bone cells. J Bone Miner Res. 1990;5:597‐608. 2166423 10.1002/jbmr.5650050609

[52] Erben RG , Bante U , Birner H , Stangassinger M . Prophylactic effects of 1,24,25‐trihydroxyvitamin D3 on ovariectomy‐induced cancellous bone loss in the rat. Calcif Tissue Int. 1997;60:434‐440. 9115161 10.1007/s002239900259

[53] Skjødt H , Gallagher JA , Beresford JN , Couch M , Poser JW , Russell RG . Vitamin D metabolites regulate osteocalcin synthesis and proliferation of human bone cells in vitro. J Endocrinol. 1985;105:391‐396. 3873510 10.1677/joe.0.1050391

[54] Clemens TL , Fraher LJ , Sandler LM , O'Riordan JL . Demonstration of circulating 1,24,25‐trihydroxyvitamin D3 in man by radioimmunoassay. Clin Endocrinol (Oxf). 1982;16:337‐343. 6284410 10.1111/j.1365-2265.1982.tb00725.x

[55] Burt LA , Kaufmann M , Jones G , Billington EO , Boyd SK & Hanley DA Is bone loss in the Calgary vitamin D study mediated by 1,25‐(OH)2D3 or its catabolite 1,24,25‐(OH)3D3? Measurements of the vitamin D metabolome. Abstract for oral presentation presented at: American Society for Bone and Mineral Research Annual Meeting; September 30–October 3, 2021; San Diego, CA.

[56] Meyer MB , Pike JW . Mechanistic homeostasis of vitamin D metabolism in the kidney through reciprocal modulation of Cyp27b1 and Cyp24a1 expression. J Steroid Biochem Mol Biol. 2020;196:105500. 31629064 10.1016/j.jsbmb.2019.105500 PMC6954286

[57] Beaudin SG , Robilotto S , Welsh J . Comparative regulation of gene expression by 1,25‐dihydroxyvitamin D3 in cells derived from normal mammary tissue and breast cancer. J Steroid Biochem Mol Biol. 2015;148:96‐102. 25239595 10.1016/j.jsbmb.2014.09.014 PMC4760099

[58] Papapoulos SE , Clemens TL , Sandler LM , Fraher LJ , Winer J , O'Riordan JL . The effect of renal function on changes in circulating concentrations of 1,25‐dihydroxycholecalciferol after an oral dose. Clin Sci (Lond). 1982;62:427‐429. 6896025 10.1042/cs0620427

[59] Gorman S , Zafir M , Lim EM , et al. High dose intramuscular vitamin D provides long‐lasting moderate increases in serum 25‐hydroxyvitamin D levels and shorter‐term changes in plasma calcium. J AOAC Int. 2017;100:1337‐1344. 28492140 10.5740/jaoacint.17-0087

[60] Bikle DD , Gee E , Halloran B , Kowalski MA , Ryzen E , Haddad G . Assessment of the free fraction of 25‐hydroxyvitamin D in serum and its regulation by albumin and the vitamin D‐binding protein. J Clin Endocrinol Metab. 1986;63:954‐959. 3745408 10.1210/jcem-63-4-954

[61] Singh RJ , Taylor RL , Reddy GS , Grebe SK . C‐3 epimers can account for a significant proportion of total circulating 25‐hydroxyvitamin D in infants, complicating accurate measurement and interpretation of vitamin D status. J Clin Endocrinol Metab. 2006;91:3055‐3061. 16720650 10.1210/jc.2006-0710

[62] Jones G , Schnoes HK , Levan L , DeLuca HF . Isolation and identification of 24‐hydroxyvitamin D_2_ and 24,25‐dihydroxyvitamin D_2_ . Arch Biochem Biophys. 1980;202:450‐457. 6970013 10.1016/0003-9861(80)90449-x

[63] Rao DS , Siu‐Caldera ML , Uskokovic MR , Horst RL , Reddy GS . Physiological significance of C‐28 hydroxylation in the metabolism of 1alpha,25‐dihydroxyvitamin D_2_ . Arch Biochem Biophys. 1999;368:319‐328. 10441383 10.1006/abbi.1999.1308

[64] DeLuca HF , Suda T , Schnoes HK , Tanaka Y , Holick MF . 25,26‐dihydroxycholecalciferol, a metabolite of vitamin D_3_ with intestinal calcium transport activity. Biochemistry. 1970;9:4776‐4780. 4319987 10.1021/bi00826a022

[65] Ponchon G , Kennan AL , DeLuca HF . "Activation" of vitamin D by the liver. J Clin Invest. 1969;48:2032‐2037. 4310770 10.1172/JCI106168 PMC297455

[66] Cheng JB , Levine MA , Bell NH , Mangelsdorf DJ , Russell DW . Genetic evidence that the human CYP2R1 enzyme is a key vitamin D 25‐hydroxylase. Proc Natl Acad Sci U S A. 2004;101:7711‐7715. 15128933 10.1073/pnas.0402490101 PMC419671

[67] Bikle DD , Halloran BP , Gee E , Ryzen E , Haddad JG . Free 25‐hydroxyvitamin D levels are normal in subjects with liver disease and reduced total 25‐hydroxyvitamin D levels. J Clin Invest. 1986;78:748‐745. 3745436 10.1172/JCI112636 PMC423667

[68] Schwartz JB , Gallagher JC , Jorde R , et al. Determination of free 25(OH)D concentrations and their relationships to total 25(OH)D in multiple clinical populations. J Clin Endocrinol Metab. 2018;103:3278‐3288. 29955795 10.1210/jc.2018-00295 PMC6126881

[69] Hoofnagle AN , Eckfeldt JH , Lutsey PL . Vitamin D‐binding protein concentrations quantified by mass spectrometry. N Engl J Med. 2015;373:1480‐1482. 10.1056/NEJMc1502602 PMC4654614 26397952

[70] Hanson C , Jones G , Lyden E , Kaufmann M , Armas L , Anderson‐Berry A . Vitamin D metabolism in the premature newborn: a randomized trial. Clin Nutr. 2016;35(4):835‐841. 26302850 10.1016/j.clnu.2015.07.023

[71] Heijboer A Abstract: economics of vitamin D testing: comparison of LC‐MS/MS and antibody‐based methods. Presented at: Third International Conference on Controversies in Vitamin D; Giustina A, Bilezikian JP, organizers. 2020; Gubbio, Italy.

[72] Graeff‐Armas LA , Kaufmann M , Lyden E , Jones G . Serum 24,25‐dihydroxyvitamin D(3) response to native vitamin D(2) and D(3) supplementation in patients with chronic kidney disease on hemodialysis. Clin Nutr. 2018;37:1041‐1045. 28506446 10.1016/j.clnu.2017.04.020

[73] Melamed ML , Chonchol M , Gutiérrez OM , et al. The role of vitamin D in CKD stages 3 to 4: report of a scientific workshop sponsored by the National Kidney Foundation. Am J Kidney Dis. 2018;72:834‐845. 30297082 10.1053/j.ajkd.2018.06.031 PMC6615058

[74] Ginsberg C , Hoofnagle AN , Katz R , et al. The vitamin D metabolite ratio is independent of vitamin D binding protein concentration. Clin Chem. 2021;67:385‐393. 33188595 10.1093/clinchem/hvaa238 PMC8880257

[75] Bishop JE , Collins ED , Okamura WH , Norman AW . Profile of ligand specificity of the vitamin D binding protein for 1 alpha,25‐dihydroxyvitamin D3 and its analogs. J Bone Miner Res. 1994;9:1277‐1288. 7976510 10.1002/jbmr.5650090818

[76] Ginsberg C , Hoofnagle AN & Katz R , et al. The vitamin D metabolite ratio is associated with changes in bone density and fracture risk in older adults. J Bone Miner Res. 2021 Aug 23. 10.1002/jbmr.4426. PMC8688212 34423858

[77] Selamet U , Katz R , Ginsberg C , et al. Serum calcitriol concentrations and kidney function decline, heart failure, and mortality in elderly community‐living adults: the health, aging, and body composition study. Am J Kidney Dis. 2018;72:419‐428. 29885925 10.1053/j.ajkd.2018.03.026 PMC6245577

[78] Jones G . Extrarenal vitamin D activation and interactions between vitamin D_2_, vitamin D_3_ and vitamin D analogs. Annu Rev Nutr. 2013;33:23‐44. 23642201 10.1146/annurev-nutr-071812-161203

[79] Molin A , Baudoin R , Kaufmann M , et al. *CYP24A1* mutations in a cohort of hypercalcemic patients: evidence for a recessive trait. J Clin Endocrinol Metab. 2015;100:E1343‐E1352. 26214117 10.1210/jc.2014-4387

